# Generation, Characterisation and Identification of Bioactive Peptides from Mesopelagic Fish Protein Hydrolysates Using In Silico and In Vitro Approaches

**DOI:** 10.3390/md22070297

**Published:** 2024-06-27

**Authors:** Maria Hayes, Azza Naik, Leticia Mora, Bruno Iñarra, Jone Ibarruri, Carlos Bald, Thibault Cariou, David Reid, Michael Gallagher, Ragnhild Dragøy, Jorge Galino, Alba Deyà, Sissel Albrektsen, Lars Thoresen, Runar G. Solstad

**Affiliations:** 1 Food BioSciences Department, Teagasc Food Research Centre, Ashtown, D15 DY05 Dublin, Ireland; azzasilotry.naik@tudublin.ie; 2 Instituto de Agroquímica y Tecnología de Alimentos (CSIC), Avenue Agustín Escardino 7, Valencia, 46980 Paterna, Spain; lemoso@iata.csic.es; 3 AZTI, Food Research, Basque Research and Technology Alliance (BRTA), Parque Tecnológico de Bizkaia, Astondo Bidea, Edificio 609, 48160 Derio, Spain; binarra@azti.es (B.I.); jibarruri@azti.es (J.I.); cbald@azti.es (C.B.); 4 Marine Institute, Fisheries and Ecosystems Advisory, Rinville, Oranmore, H91 R673 Co. Galway, Irelanddavid.reid@ices.dk (D.R.); 5 Bord Iascaigh Mhara, The Pier, Killybegs, F94 P8YP Co. Donegal, Ireland; 6 Department of Marine Biotechnology, Muninbakken 9-13, 9019 Tromsø, Norway; 7 Aker BioMarine, Oksenøyveien 10, 1366 Lysaker, Norway; 8 Health and Biomedicine Department, Leitat Technological Center, Carrer de la Innovació, 2, 08225 Terrassa, Spain; 9 Department of Nutrition and Feed Technology, Nofima AS, P.O. 5844 Oasen, 5828 Bergen, Norway

**Keywords:** peptides, anti-inflammatory, heart health, anti-diabetic, type-2-diabetes, in silico analysis, mesopelagic fish, hydrolysates, circular economy

## Abstract

This study generated bioactive hydrolysates using the enzyme Alcalase and autolysis from mesopelagic fish, including *Maurolicus muelleri* and *Benthosema glaciale*. Generated hydrolysates were investigated for their bioactivities using in vitro bioassays, and bioactive peptides were identified using mass spectrometry in active hydrolysates with cyclooxygenase, dipeptidyl peptidase IV and antioxidant activities. In silico analysis was employed to rank identified peptide sequences in terms of overall bioactivity using programmes including Peptide Ranker, PrepAIP, Umami-MRNN and AntiDMPpred. Seven peptides predicted to have anti-inflammatory, anti-type 2 diabetes or Umami potential using in silico strategies were chemically synthesised, and their anti-inflammatory activities were confirmed using in vitro bioassays with COX-1 and COX-2 enzymes. The peptide QCPLHRPWAL inhibited COX-1 and COX-2 by 82.90% (+/−0.54) and 53.84%, respectively, and had a selectivity index greater than 10. This peptide warrants further research as a novel anti-inflammatory/pain relief peptide. Other peptides with DPP-IV inhibitory and Umami flavours were identified. These offer potential for use as functional foods or topical agents to prevent pain and inflammation.

## 1. Introduction

Mesopelagic fish occur in the twilight zone at depths of between 200–1000 m and are largely underexplored and not exploited currently as food, feed or as a source of bioactive compounds. Mesopelagic species include *Maurolicus muelleri* (Mueller’s Pearlside) and *Meganyctiphanes norvegica* (Northern Krill) [1]. Recent work in the MEESO and SUMMER EU H2020 projects identified Angiotensin-1-converting enzymes (ACE-1) inhibitory, Dipeptidyl peptidase IV (DPP-IV) inhibitory and antimicrobial activities in hydrolysates and extracts generated from mixed mesopelagic fish trawls [2]. Additional studies by Aliyu and colleagues (2022) identified the antioxidant potential of hydrolysates generated from *M. muelleri* and Krill [3]. More recently, Medina and colleagues looked at the onboard processing of mesopelagic species, including *M. muelleri* and *Benthosema glaciale*, as a source of dietary lipids [4,5].

Hydrolysis of difficult-to-process biomass with enzymes is a useful approach for the generation of high-quality protein products. Enzymes impart selectivity and specificity to products, and hydrolysis can add value to proteins by increasing the digestibility, bioavailability and bioactivities of the protein biomass by the creation of bioactive peptides. Additionally, hydrolysis can reduce the potential of proteins to cause allergy [6,7]. Bioactive peptides are sequences between 2–30 amino acids in length that may be derived from parent proteins using hydrolysis and have several known bioactivities [8]. A database of bioactive peptides known as BIOPEP-UWM [9] contains over 4800 bioactive peptides identified from natural resources. Bioactivities ordinarily reported in this database include ACE-1 inhibitors, DPP-IV inhibitors, antioxidative and antimicrobial peptides. Bioactive and functional food peptides can positively affect the regulation of an individual’s health by preventing various ailments and by improving medical conditions related to lifestyle, metabolism, and immunity. Several therapeutic peptides are approved for use in the mitigation of blood disorders and diseases associated with metabolic syndrome, such as type 2 diabetes (T2D). For example, the drug Liraglutide (Victoza)—a glucagon-like peptide-1 receptor (GLP-1R) agonist—is approved for the treatment of T2D and is a top-selling drug produced by Novo Nordisk [10]. Other peptide therapies include Goserelin [11,12].

Chronic inflammation is a prolonged, over-reactive immune system response, and if it goes unchecked, it keeps the body in a constant state of stress and high alert, resulting in disease conditions like arthritis, psoriasis and irritable bowel disease (IBD) [13,14,15,16]. Drugs, including ibuprofen and aspirin, act on targets for inflammation and pain, including cyclooxygenase (COX) enzymes, to reduce pain and treat chronic inflammation. They are non-steroidal anti-inflammatory drugs (NSAIDs) that are highly effective, but their long-term use is linked to toxicity, high blood pressure and hepatic issues [17,18,19]. Worldwide, alternatives to NSAIDs are sought from natural sources. Cyclooxygenase (COX; E.C. 1.14. 99.1) enzymes convert arachidonic acid into inflammatory prostaglandins (PGs) and are targets for anti-pain and anti-inflammatory bioactives and some patents concerning food-derived ingredients and their use for preventing pain and inflammation exist including berry extracts [20]. Monoacylglycerol lipase (MAGL; EC 3.1. 1.23) is an endocannabinoid-degrading enzyme converting 2-arachidonoylglycerol (2-AG), an endogenous ligand for the cannabinoid receptors CB1 and CB2, into arachidonic acid. MAGL terminates 2-AG signaling and is the major source of arachidonic acid and proinflammatory prostaglandins in the liver or in the brain. It is considered an anti-inflammatory target [21,22,23,24,25,26].

Natural COX and MAGL inhibitory compounds exhibiting anti-inflammatory activity, which can be included in functional foods or within nutraceutical formulations, are a potentially better alternative to synthetic drugs for the prevention and treatment of inflammation and pain. Renin and DPP-IV inhibitors can help to modulate innate and adaptive immunity. Inhibition of DPP-IV has known beneficial effects concerning glycemic control in cardiovascular disease and renal issues. In addition, DPP-IV inhibitors can help reduce weight in obese individuals.

The scientific purpose of this work was the generation, characterisation, synthesis and confirmation of bioactivities, specifically, anti-pain and anti-inflammatory bioactivities derived from the mesopelagic fish, *M. muelleri* using Alcalase hydrolysis. Targets for identified peptides include COX-1, COX-2 and MAGL enzymes. Peptides were identified using mass spectrometry, and in silico analysis was used to select peptides for chemical synthesis. Eight peptides were made and assessed for their COX-1, COX-2 and MAGL inhibitory activities in vitro. COX-1, COX-2 and MAGL EC_50_ values were assigned to three novel peptides. Novel peptides discovered and not found in databases like BIOPEP-UWM included the peptides with amino acid sequences QCPLRPWAL, NVGEVVCIFLTALGLPEALI, and FDFLPM derived from *M. muelleri* proteins.

## 2. Results and Discussion

### 2.1. Hydrolysate Generation from Irish and Norwegian Mesopelagic Trawls and Proximate Compositional Analysis of Resulting Hydrolysates

The degree of hydrolysis for Irish hydrolysate samples made using Alcalase^®^ applied to *Notocopelus elongtus kroyeri*, *Benthosema glaciale* and *Maurolicus muelleri* as well as Blue whiting species ranged from 50–70%. Following the generation of hydrolysates and stabilisation using freeze-drying, hydrolysates were characterised for their protein, ash, lipid and moisture content using AOAC methods for protein, ash and lipid content, as described previously [27]. The protein content of generated hydrolysates ranged from 14.04% (hydrolysate generated from *Notocopelus elongtus kroyeri*) to 18.02% (hydrolysate generated from *M. muelleri*). The ash content of hydrolysates generated from Irish mesopelagic trawls using Alcalase^®^ was between two (*Notocopelus elongtus kroyeri*) to 3.8% (*M. muelleri*), and the lipid content ranged from 0.46% (hydrolysate of *Maurolicus muelleri* with Alcalase^®^) to 9.92% (*Benthosema glaciale* hydrolysate with Alcalase^®^) of dry weight.

### 2.2. Hydrolysate Generation from Spanish Mesopelagic Fish Trawl

**Figure 1:**
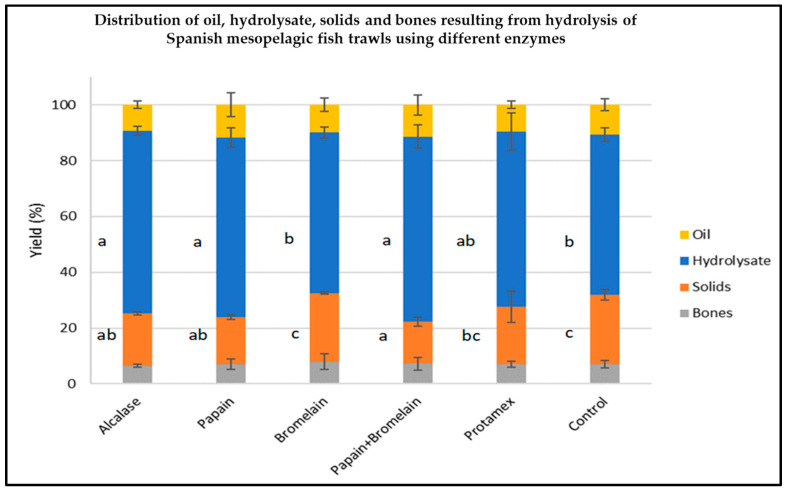
Distribution of oil, hydrolysate, solids and bones generated using different enzyme combinations applied to Spanish mesopelagic trawls consisting of the species *M. muelleri.* Error bars represent SD (*n* = 3). Different letters indicate where a significant difference exists between samples at 95% confidence.

The yields of solids, oil, hydrolysates and bones generated using different enzymes applied to Spanish mesopelagic trawls consisting of *M. muelleri* are shown in Figure 1. The greatest yield of hydrolysate resulted from the use of Alcalase and Papain applied to the mesopelagic biomass (*M. muelleri* solely) and the combination of Papain and Bromelain applied to the same biomass. The lowest hydrolysate yields were obtained using Bromelain, and no added enzyme (control, with only endogenous enzymes) was applied to *M. muelleri* biomass. The degree of hydrolysis observed for the hydrolysates generated from *M. muelleri* using different commercial enzymes ranged from 50–60%.

### 2.3. Cyclooxygenase (COX) Enzyme Inhibition by Generated Hydrolysates

**Figure 2:**
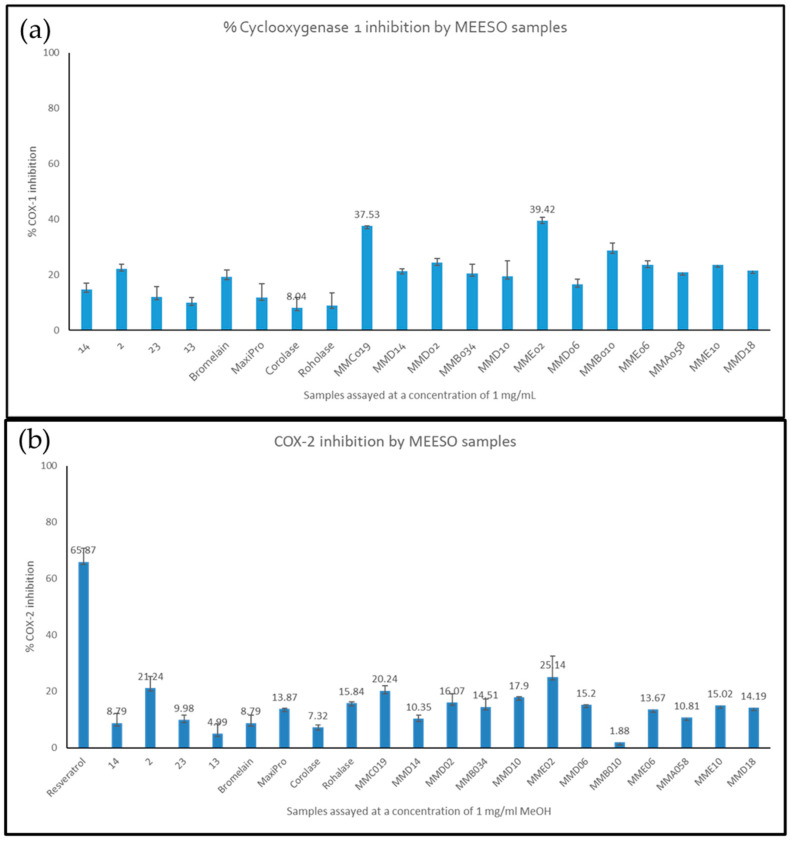
(**a**) COX-1 inhibition by whole mesopelagic hydrolysates generated with different proteolytic enzymes. Samples were assayed at a concentration of 1 mg/mL and compared to a commercial control. Assays were performed in triplicate (*n* = 9). *(***b**) COX-2 inhibition by whole mesopelagic hydrolysates generated with different proteolytic enzymes. Samples were assayed at a concentration of 1 mg/mL and compared to a commercial control, Resveratrol. Assays were performed in triplicate (*n* = 9). Irish hydrolysates: 14 corresponds to CE21004 Haul4 (Code 14) *Benthosema glaciale* biomass hydrolysed with Alcalase^®^; 2 corresponds to CE21004 Haul2 (Code 2) *Notocopelus elongtus kroyeri* hydrolysed with Alcalase^®^; 23 corresponds to CE21009 Haul23 *Maurolicus muelleri* biomass hydrolysed with Alcalase^®^; 13 corresponds to CE21004 Haul13 (Code 13) *Maurolicus muelleri* mixed biomass hydrolysed with Alcalase^®^. Norwegian hydrolysates: Bromelain corresponds to *M. muelleri* biomass hydrolysed with Bromelain; MaxiPro corresponds to *M. muelleri biomass* hydrolysed with MaxiPro enzymes; Corolase corresponds to *M. muelleri* biomass hydrolysed with Corolase and Roholase corresponds to *M. muelleri* hydrolysed with the Roholase enzyme. Spanish hydrolysates: MMD02 and MME02 correspond to *M. muelleri* hydrolysed with Alcalase 2.4 LG; MMD06 and MME06 correspond to *M. muelleri* hydrolysed with Papain; MMD10 and MME10 correspond to *M. muelleri* hydrolysed with Bromelain; MMB010 and MMD18 correspond to *M. muelleri* hydrolysed with Protamex; MMA058 and MMD14 correspond to *M. muelleri* hydrolysed with Papain and Bromelain and MMB034 and MMC019 correspond to hydrolysates generated from *M. muelleri* using endogenous enzymes.

Freeze-dried Irish, Norwegian and Spanish hydrolysates generated from mesopelagic species with different enzymes were assessed at concentrations of 1 mg/mL in dimethyl sulfoxide (DMSO) to determine their potential to prevent pain and inflammation via the COX-1 and COX-2 inhibition pathways (Figure 2a,b). *M. muelleri* hydrolysed with Alcalase 2.4 LG (MME02) and *M. muelleri* hydrolysed by endogenous enzymes (MMC019) were found to inhibit COX-1 enzyme by 39.42% and 37.53% when assayed at concentrations of 1 mg/mL compared to a positive control. The same extracts were found to inhibit COX-2 by 25.14% and 20.14%, respectively, compared to Resveratrol, which inhibited COX-2 by 65.87% (the positive control). In addition, the Irish hydrolysate generated from *Benthosema glaciale* biomass with Alcalase^®^ (labelled 2 corresponds to CE21004 Haul2) inhibited both COX-1 and COX-2 by 20–22% when assayed at 1 mg/mL.

### 2.4. Monoacylglycerol Lipase (MAGL) Enzyme Inhibition by Generated Hydrolysates

MAGL is a serine hydrolase that hydrolyses monoglycerides into glycerol and fatty acids. It links the endocannabinoid and eicosanoid systems through the degradation of endocannabinoid 2-arachidaoylglycerol into arachidonic acid, the precursor of prostaglandins and other inflammatory mediators. MAGL inhibitors are known as anti-nociceptive, anxiolytic and anti-inflammatory agents [28]. Irish mesopelagic species hydrolysates were assessed for their ability to inhibit the MAGL enzyme. *M. muelleri* biomass hydrolysed with Alcalase^®^ (labelled sample 23) and passed through a 3-kDa molecular weight cut-off (MWCO) filter inhibited MAGL by 63.19% when assayed at a concentration of 1 mg/mL in 1% DMSO and water. The positive control, the compound JZL 195, inhibited MAGL by 98.16% when assayed at a concentration of 4.4 µM. All other extracts did not significantly inhibit MAGL when assayed at a similar concentration. These hydrolysates were selected for further characterisation using mass spectrometry and in silico analysis.

### 2.5. 2,2′-Azino-bis-3-ethylbenzthiazoline-6-sulphonic Acid Total Antioxidant Capacity (ABTS) of Mesopelagic Hydrolysates

**Figure 3:**
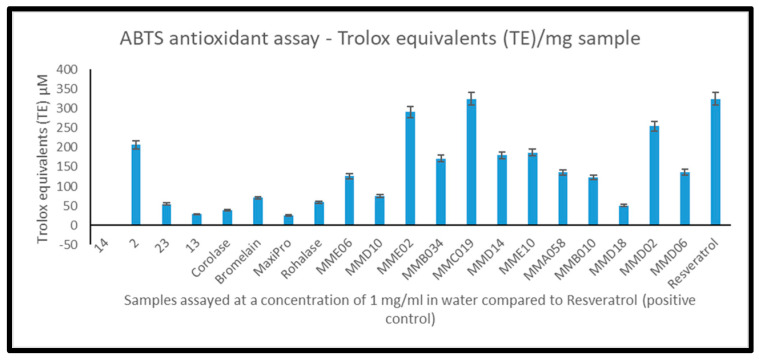
Antioxidant activity of whole, mesopelagic hydrolysates generated with different proteolytic enzymes. Samples were assayed using the ABTS antioxidant assay at a concentration of 1 mg/mL and compared to a commercial control Resveratrol. Assays were performed in triplicate (*n* = 9). Samples are represented by code, as shown in Figure 2.

The hydrolysates generated from Spanish mesopelagic fish, *M. muelleri*, hydrolysed with Alcalase 2.4 L (MME02 and MMD02) and *M. muelleri* hydrolysed by endogenous enzymes (MMC019) displayed antioxidant activities as well as the Irish hydrolysate generated from *Benthosema glaciale* hydrolysed with Alcalase^®^ (sample 2, Figure 3). These hydrolysates quenched the ABTS radical maximally compared to the positive control Resveratrol^®^ and had ABTS-TAC values of 325 (±24.79) µM TE/mg; 330 µM TE/mg and 210 µM TE/mg, respectively. Resveratrol quenched the radical by 330 µM TE/mg (Figure 3). These hydrolysates were selected for further characterisation using mass spectrometry and in silico analysis.

### 2.6. Angiotensin-1-Converting Enzyme (ACE-1) and Renin Inhibition by Mesopelagic Hydrolysates

**Figure 4:**
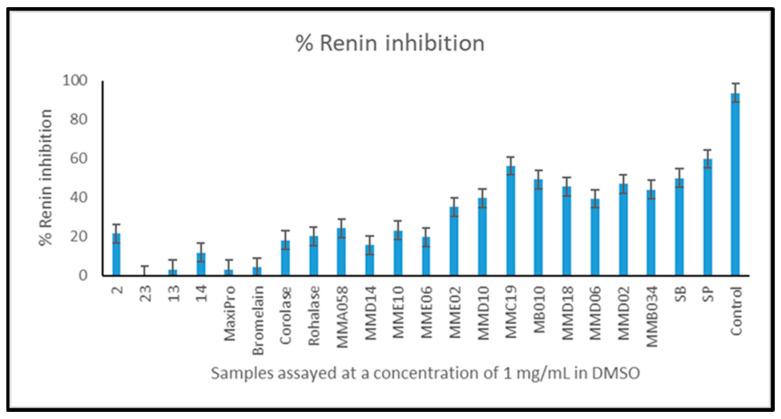
Renin inhibition by whole, mesopelagic hydrolysates generated with different proteolytic enzymes. Samples were assayed using the renin inhibition assay method described in the materials and methods section at a concentration of 1 mg/mL and compared to commercial and internal controls SB, SP and Captopril. Assays were performed in triplicate (*n* = 9). Samples are represented by codes, as shown in Figure 2.

All hydrolysates generated from Irish mesopelagic fish hauls, including *Benthosema glaciale* biomass hydrolysed with Alcalase^®^; *Notocopelus elongtus kroyeri* hydrolysed with Alcalase^®^; *M.muelleri* biomass hydrolysed with Alcalase^®^ and *M. muelleri* mixed biomass hydrolysed with Alcalase^®^ inhibited ACE-1 by >95% when assayed at a concentration of 1 mg/mL compared to the positive control, assayed at a concentration of 0.5 mg/mL that inhibited ACE-1 by 100%. Specifically, for *M. muelleri* biomass hydrolysed with Alcalase^®^, the ACE-I IC50 was 27 µg/mL. These results are quite promising compared to other published ACE inhibition bioactivities of marine hydrolysates as, for instance, salmon protein hydrolysate (IC50 of 20–140 µg/mL), manila clams (IC50 of 420 µg/mL) or tuna frame protein hydrolysate (IC50 of 2 mg/mL). Inhibition of the enzyme Renin by mesopelagic hydrolysates is shown in Figure 4. Mesopelagic hydrolysates inhibited Renin by between 20–59% (Figure 4) when assayed at a concentration of 1 mg/mL. MMC19 (endogenous enzymes) and MMD02 (Alcalase 2.4 LG) gave results comparable to commercial controls SB and SP.

### 2.7. Dipeptidyl Peptidase IV Inhibition by Mesopelagic Hydrolysates

Generated mesopelagic hydrolysates were assessed for their ability to inhibit DPP-IV at concentrations of 1 mg/mL using Sitagliptin as a positive control. Only the extract generated from *Benthosema glaciale* biomass using Alcalase^®^ was found to inhibit DPP-IV by 17.33% when assayed at a concentration of 1 mg/mL compared to the positive control Sitagliptin that inhibited DPP-IV by 68.79%. The active hydrolysate was assessed further using MS and in silico analysis.

### 2.8. Identification of Bioactive Peptides Using Mass Spectrometry and In Silico Analysis

Hydrolysates identified as having bioactivities using in vitro bioassays described in Section 4 were characterised following hydrolysate clean-up using MS. Bioactive hydrolysates included CE21009 Haul 23 *Maurolicus muelleri* (Code 23) Alcalase hydrolysate, an Irish sample; *Maurolicus muelleri* (MMC019) Endogenous enzyme autolysis (Spanish sample), spray dried and *Maurolicus muelleri* (MME02—Spanish haul) alcalase hydrolysate. 1724, 95 and 182 peptide sequences were identified from each hydrolysate, respectively. These peptides had homology with several different proteins of different origins. Following identification, peptides were ranked in terms of their potential to be bioactive using an in silico approach expanded upon from previously published work by our group [29,30]. A peptide ranker was used to predict the potential bioactivity of peptide sequences based on amino acid charge and peptide structure, as described previously [31]. The freely available programme PreAIP available at http://kurata14.bio.kyutech.ac.jp/PreAIP/ (accessed on 23 January 2024) [32] was used to predict the collective anti-inflammatory activity of peptides identified in each hydrolysate and anti-inflammatory potential of individual peptides. The Predictor of Anti-Inflammatory Peptides (PreAIP) programme predicts bioactivity, specifically anti-inflammatory bioactivity, by looking at the primary sequence, as well as evolutionary and structural information of the peptide, through a random forest classifier [32]. Scores ≥ 0.468 indicate high confidence that a peptide is anti-inflammatory. Scores between 0.468 and ≥ 0.388 indicate medium confidence that a peptide is anti-inflammatory, while a score of 0.388 to 0.342 indicates that the peptide has a low probability of having anti-inflammatory activity [32]. A search for sequenced peptides in the database BIOPEP-UWM was used to confirm the novel nature of the mesopelagic fish species-derived peptides, and Umami-MRNN https://umami-mrnn.herokuapp.com/ (accessed on 23 January 2024) [33] was used to predict if peptides would impart umami flavour or not. The database service AntiDMPpred, available at http://i.uestc.edu.cn/AntiDMPpred/cgi-bin/AntiDMPpred.pl (accessed on 23 January 2024), was used to identify the anti-diabetes type 2 activity of individual peptides [34]. AntiDMPpred is a model that predicts the potential of peptides to be anti-diabetic from sequence information. The threshold of the probability value to distinguish ant-diabetic peptides from non-anti-diabetic peptides ranges from 0–1. The closer the value is to 1, the higher the probability that the peptide is anti-diabetic [34]. The 10 most bioactive peptides found in each hydrolysate are shown in Table 1. The hydrolysates generated from *Maurolicus muelleri* using Alcalase (Irish mesopelagic fish sample) were found to contain peptides from several different proteins. Only three peptide sequences in this hydrolysate predicted to be anti-inflammatory and to impart Umami flavour were derived from fish proteins. These include the peptide FDAFLPM with a Peptide Ranker score of 0.955, predicted to be a medium anti-inflammatory agent and with no Umami potential derived from Thin-lipped mullet proteins. The peptide QCPLHRPWAL identified as having high potential anti-inflammatory activity (PreAIP RF value of 0.499) has amino acid homology with a protein derived from Atlantic salmon (*Salmo salar*) and the peptide NVGEVVCIFLTAALGLPEALI predicted to be highly anti-inflammatory (PreAIP RF value of 0.612) and also predicted to have anti-diabetic potential using AntiDMPpred (probability value of 0.8) has sequence homology with a protein from *Makaira nigricans* commonly known as Atlantic Blue Marlin. The anti-inflammatory potential of the 10 most bioactive peptides generated in this hydrolysate was also predicted to be significant with a PreAIP RF value of 0.482 (high confidence anti-inflammatory). The hydrolysate generated from *Maurolicus muelleri* (MMC019) with only endogenous enzyme autolysis (Spanish sample) and subsequently spray dried also has predicted anti-inflammatory activities with a combined PreAIP value of 0.514 for the 10 most bioactive active peptides identified using Peptide Ranker. Individually, these peptides had predicted anti-inflammatory potential values ranging from 0.540 (identified using PreAIP RF) for the peptide IAGFEIFDFNSLEQLC, derived from fish proteins from the species *Cypinus carpio* (Common Carp) to 0.284 (not anti-inflammatory according to PreAIP RF) for the peptide GFAGDDAPR derived from fish proteins identified with homology to proteins derived from *Takifuga rubipes* (Japanese Puffer Fish). The hydrolysate generated from *Maurolicus muelleri* (MME02—Spanish haul) using Alcalase enzyme had a medium confidence AIP with a PreAIP RF value of 0.446. The peptide SDNAYQFMLT, identified as having sequence homology to proteins derived from Common Carp, is predicted to have medium anti-inflammatory activity with a PreAIP value of 0.412. In addition, this peptide was predicted to impart an Umami flavour with a Umami-predicted threshold of 34.065384 mmol/L (Table 1).

### 2.9. Chemical Synthesis and Confirmation of Anti-Inflammatory Activity of Peptides Using In Vitro COX and MAGL Inhibition Bioassays

Peptide IAGFEIFDFNSLEQLC was predicted to have anti-inflammatory and Umami imparting bioactivities and with sequence homology to a protein derived from *Cypinus carpio* (Common Carp); QCPLHRPWAL with homology to a protein from Atlantic salmon and predicted anti-inflammatory activities, SDNAYQFMLT with predicted anti-inflammatory activity and sequence homology to a fish protein from Common carp, as well as the peptides PSRILYG, FDFLPM, GFNPPDLDIM and GFAGDDAPR were chemically synthesised by GenScript Biotech (Leiden, The Netherlands). GenScript also verified the purity of the peptides by analytical RP-HPLC–MS. COX-1 and COX-2 percentage (%) inhibition values, COX half-maximal Inhibitory Concentration (IC_50_) values and the selectivity index of selected peptides were determined for these synthesised peptides and values compared to the control, a commercially available COX inhibitor drug Diclofenac. The peptide IAGFEIFDFNSLEQLC inhibited COX-1 by 53.26% (+/−5.26) when assayed at a concentration of 1 mg/mL with a COX-1 IC_50_ value of 189.76 µg/mL (102.79 µM). This peptide inhibited COX-2 by 47.46% when assessed at a concentration of 1 mg/mL with a COX-2 IC_50_ value of 366.48 µM and a selectivity index value of 0.52. Peptide QCPLHRPWAL inhibited COX-1 by 82.90% (+/−0.54) with a COX-1 IC_50_ value of 2386.64 µM. This peptide inhibited COX-2 by 53.84% when assayed at a concentration of 1 mg/mL and displayed a COX-2 IC_50_ value of 220.66 µM with a selectivity index value of 10.81. Selectivity index (SI) refers to the ratio of the toxic concentration of a sample against its effective bioactive concentration. An SI value ≥ 10 shows the potential that the peptide is non-toxic but bioactive and warrants further investigation [35]. Peptide SDNAYQFMLT inhibited COX-1 by 53.72% when assayed at a concentration of 0.5 mg/mL, and peptide FDAFLPM inhibited COX-1 by 34.49% at the same concentration. The peptide NVGEVVCIFLTAALGLPEALI derived from Irish *M. muelleri* hauls and hydrolysed with Alcalase^®^ was also chemically synthesised and assessed for its ability to inhibit the MAGL enzyme. This peptide inhibited MAGL by 53.05% (+/−5.25) when assayed at a concentration of 0.5 mg/mL compared to the positive control, JZL 195 and had a MAGL IC_50_ value of 13.61 µg/mL (6.35 µM).

Recently, Rivera-Jiménez and colleagues collated an excellent review concerning peptides and protein hydrolysates exhibiting anti-inflammatory activities and detailed different sources, structural features and modulation mechanisms for the limited number of anti-inflammatory hydrolysates from fish that are reported in the literature [36]. The review details peptides of fish origin derived from salmon skin and pectoral fin hydrolysates of the species *Salmo salar*, as well as peptides from hydrolysates of herring millet, mollusc, abalone and oyster. All listed fish-derived anti-inflammatory peptides regulated inflammation by inhibition of nitric oxide (NO) production and inhibition of IL-6 and TNF-α [36]. Peptides identified from bivalve visceral mass and salmon bones were COX-2 inhibitors with inhibition values ranging from 45–58% and NO IC_50_ values of 54.07 µg ml^−1^ reported for the peptide PFNEGTFAAS derived from mollusc abalone [37]. No anti-inflammatory peptides that work by inhibition of MAGL are reported in this paper or in databases like BIOPEP-UWM. In addition, confirmation of anti-inflammatory activities by synthesis of COX-2 peptides is not reported in this review.

Herein, we have identified novel bioactive peptides with unique amino acid sequences derived from mesopelagic fish *M. muelleri* and *Benthosema* sp. hydrolysed with the enzyme Alcalase. In many cases, an enzyme-to-substrate ratio of 1:100 (*v*:*w*) and a hydrolysis time of 3 h were used to obtain the hydrolysates. Peptides with COX-1, COX-2 and MAGL inhibitory activities were identified for hydrolysates generated under these conditions. The enzyme inhibitory effect of seven peptides identified from these hydrolysates was determined. All synthesised peptides inhibited COX-1 and COX-2 by between 34–54% when assayed at 1 mg/mL concentrations, and peptide IAGFEIFDFNSLEQLC had COX-1 and COX-2 values of 102.79 µM and 366 µM, respectively. Only one peptide, peptide NVGEVVCIFLTAALGLPEALI derived from Irish *M. muelleri* hydrolysed with Alcalase, inhibited the enzyme MAGL and had an MAGL IC_50_ value of 6.35 µM. The COX and MAGL inhibitory potential of identified peptides compare favourably with previously identified anti-inflammatory peptides like LREMLSTMCTARGA, AVGPGPRG and VPWGPWPKG from a bromelain hydrolysate of sea cucumber that inhibited NO in murine macrophages with reported NO IC_50_ values of 572.096 mg/mL and 674.435 mg/mL respectively [38]. Previously, anti-inflammatory peptides from pure amaranth with the peptide sequences HGSEPFGPR and RPRYPWRYT were found to reduce LOX-1 expression, and these peptides had IC_50_ values comparable to the IC_50_ values determined against COX-1 and COX-2 for the mesopelagic derived peptides [39]. It is possible that different peptides would result if the conditions of hydrolysis were changed from 1:100 (*v*:*w*) enzyme: substrate to 1:10 (*v*:*w*) and if the duration of hydrolysis was lessened. However, optimisation of the hydrolysis process was not the focus of this study. Previously, Shahi and colleagues investigated the effect of enzymatic hydrolysis times (40–240 min) with Alcalase and pancreatin in enzyme-substrate ratio (2% *w*/*w*) on the hydrolysis degree and antioxidant properties of bioactive peptides from defatted *Black cumin* press cake. Hydrolysis is normally enhanced by increasing the process time. A time of 3 h (240 min) usually produces peptides with a molecular weight of <10 kDa, and this can impact the bioactivity of peptides [40].The peptides identified here are not as active as natural products derived from Chinese herbs, such as the compounds Acetyl-11-keto-β-boswellic acid, β-boswellic acid, acetyl-α-boswellic acid, acetyl-β-boswellic acid, known COX-1 selective inhibitors with IC_50_ values of approximately 10 μM. The Chinese medicine-derived compounds Senkyunolide O and cryptotanshinone, known COX-2 selective inhibitors, were reported to have COX2- IC_50_ values of 5 μM and 22 μM, respectively, previously [41]. QCPLHRPWAL inhibited COX-1 by 82.90% (+/−0.54) and COX-2 by 53.84% with a selectivity index value of 10.81. This peptide, along with peptide NVGEVVCIFLTALGLPEALI, warrants further examination in in vivo trials based on their IC_50_ values, novelty and selectivity index values. The whole hydrolysates generated from mesopelagic fish species, independently, did not display COX-1 or COX-2 inhibitory activities greater than 50% when assayed at concentrations of 1 mg/mL. However, In silico analysis work applied to the generated hydrolysates using PreAIP suggests that they, too, are anti-inflammatory, and this activity could be enhanced by generating molecular weight cut-off (MWCO) fractions to enrich the listed bioactive peptides in Table 1. The *Maurolicus muelleri* Alcalase hydrolysate inhibited MAGL when assayed at a concentration of 1 mg/mL by 62% and was the only hydrolysate to do so. The MAGL inhibitory peptide NVGEVVCIFLTAALGLPEALI had an MAGL IC_50_ value of 6.35 µM. No fish derived peptide inhibitors of MAGL are reported in the literature or databases like BIOPEP-UWM currently. The discovery of this peptide and its IC_50_ value are novel and highly active. Previously, Jha and colleagues reported the discovery of two compounds with promising MAGL inhibitory potency as they had IC_50_ values below 50 µM [42]. In addition, this peptide was found to have the potential to impact the development of type 2 diabetes, as shown in Table 1, where it was the only peptide identified as having anti-diabetic potential using in silico analysis with the programme AntiDMPred. In addition, this peptide had an umami threshold value of 19.89 mmol/L. A typical Umami peptide such as –Lys-Gly-Asp-Glu-Glu-Ser-Leu-Ala (HKGDFFSLA) derived from beef gravy, has an umami threshold value of 36.73 mmol/L when assessed using umami-MRNN [43]. Indeed, several of the peptides identified in the mesopelagic hydrolysates that are bioactive and have predicted anti-inflammatory and anti-diabetic activity also are predicted to impart umami flavours with values predicted for different peptides of between 1.68 mmol/L for peptide GFAGDDAPR to 37.67 mmol/L for peptide LACNCNLHARRCRFNM. Umami, derived from the Japanese language, refers to a “delicious savoury taste” and is the fifth primary taste sense [44]. In the development of pet foods, palatability is important because if dogs/cats do not eat nutritionally balanced pet food, they will lack a healthy diet. Cats generally like meaty, Umami flavours as they are obligate carnivores, and research has shown that they possess glutamate receptors [45]. Identified novel peptides have the potential for use in topical applications, functional foods or supplements; however, in vivo confirmation of bioactivities is required.

## 3. Materials and Methods

### 3.1. Supply and Processing of Raw Materials

The mesopelagic samples used in this work were supplied from different experimental cruises carried out by Norwegian (Pelagia, Norway), Irish (Marine Institute, Galway, Ireland) or Spanish (AZTI and the Spanish Institute of Oceanography (IEO), Madrid, Spain) partners, respectively as part of the MEESO (EU Horizon 2020; grant agreement No 817669) and SUMMER (EU Horizon 2020; grant agreement No 817806). Irish and Norwegian samples were collected as part of the Western European Shelf Pelagic Acoustic Survey (WESPAS) and Blue Whiting oceanographic campaign conducted in March, April and May 2021. Samples supplied and subsequently analysed by AZTI were composed exclusively of *Maurolicus muelleri* species that resulted from two JUVENA oceanographic campaigns conducted in September 2019 and 2020 carried out by AZTI and the Spanish Institute of Oceanography (IEO) for the estimation of the stock of the anchovy’s fisheries in the Bay of Biscay. The samples were frozen and glazed on board the ships, and different quantities of biological material were kept in separate bags for compositional analysis and hydrolysis assays. All samples were stored at −18 °C until processing.

### 3.2. Enzymatic Hydrolysis of Irish, Norwegian and Spanish Samples

Hydrolysis of Irish mesopelagic species (Haul codes 2, 13, 14, and 23) was performed independently using a New Brunswick 1 L bioreactor (Mason Technology, Dublin, Ireland) with temperature, stirring (RPM) and pH control. The enzyme Alcalase CLEA was added individually at an enzyme-to-substrate ratio of 1:100 (*v*:*w*) (1%), and the optimum conditions for the enzyme were maintained–temperature 60 °C, pH 7 (maintained using 1 M NaOH or 1 M HCL) and rpm 250 for 4 h. The enzyme was deactivated by heating the hydrolysates to 95 °C for 15 min in a water bath (Grant JB Aqua 12, Grant Instruments, Cambridgeshire, UK). Hydrolysis was performed in triplicate. Hydrolysates were generated from mesopelagic species caught in Norwegian waters using enzymes including Bromelain, Roholase, Corolase and MaxiPro using conditions optimised for each enzyme at Nofima. These hydrolysates were assessed for bioactivities, as shown in later sections. The hydrolysis processes were performed at a laboratory scale using Symphony 7100 Bathless Dissolution Distek equipment (Distek Inc., North Brunswick, NJ, USA). In all the processes, the temperature, time and stir speed were controlled and monitored. Following hydrolysis, enzymes were inactivated by heat treatment at 95 °C for a period of 15 min. Hydrolysis of Spanish trawl samples was performed at a laboratory scale using Symphony 7100 Bathless Dissolution Distek equipment (Distek Inc., North Brunswick, NJ, USA). In all the processes, the temperature, time and stir speed were controlled and monitored. Following hydrolysis, enzymes were inactivated by heat treatment at 95 °C for a period of 15 min. Bioreactor contents were sieved to separate the bones and centrifuged (2650× *g*; 15 min; ambient temperature) to separate 3 different layers: (1) an oil-water emulsion (top-layer), (2) a water-based fraction (the protein hydrolysate—the middle layer) and (3) a solid pellet (bottom layer). Hydrolysates were freeze-dried for subsequent processing and analysis. Four different enzymes, with different enzymatic activity, were tested to produce protein hydrolysates: Protamex^®^ (NOVOZYMES, Bagsvaerd, DK), a broad-spectrum endo-protease; Alcalase^®^ 2.4 L FG (NOVOZYMES, Bagsvaerd, DK), an endo-protease of the serine-type; Papain P144GL/100 (90–110 u/g) (Biocatalysts Ltd., Cardiff, UK); Bromelain P523MDP/2000 (1,800–2,200GDU/g) (Biocatalysts Ltd., Cardiff, UK) and a combination of Papain plus Bromelain. A control (CTR) was added to the trials, which were carried out without enzyme addition at pH 6.0, 50 °C and 3 h. The pH of each run of the experimental design was controlled manually and adjusted with NaOH 1 M in a final volume of 500 mL (250 g of fish + 250 g water). All the processes were carried out with 1% enzyme (protein-based) for 3 h at 250 rpm and at the optimum pH and temperature of each enzyme or enzyme combination.

### 3.3. Hydrolysate Enrichment Using Molecular Weight Cut-Off (MWCO) Filtration

Following hydrolysis of Irish mesopelagic species with Alcalase CLEA, 3-kDa fractions were generated using molecular weight cut-off (MWCO) filters 3-kDa (Millipore Carrigaline, Co. Cork, Ireland) with the Prep/Scale™^-^TFF Cartridge Holder system where pressure was maintained at 20 psi at room temperature for 1 h. These fractions were labelled as 3-kDa permeates. All fractions were frozen, freeze-dried and stored at −20 °C until further use. Hydrolysates were freeze-dried for subsequent processing and analysis. Hydrolysates and permeates were freeze-dried in an industrial-scale freeze-drier FD 80 model at Teagasc (Cuddon Engineering, Blenheim, New Zealand).

### 3.4. Proximate Compositional Analysis of Mesopelagic Species

The total protein content of whole mesopelagic fish, hydrolysates and permeates was determined using the LECO FP328 Nitrogen determination method (LECO Corporation, Joseph, MI, USA) based on the Dumas method of nitrogen determination and according to the AOAC method 992.15, 1990 [30]. Ash content was determined using a carbolite muffle furnace according to the method of Pearson. The lipid content was quantified using the AOAC Method 2008.06 with an Oracle Rapid NMR Fat Analyzer (CEM Corporation, Matthews, NC, USA) as described previously [46,47].

### 3.5. Bioactivity Assessments of Hydrolysates and Peptides with In Vitro Screening Assays

#### 3.5.1. Cyclooxygenase (COX; EC E.C. 1.14. 99.1) Inhibition COX-1 and COX-2

The anti-pain and anti-inflammatory bioactivities of generated mesopelagic hydrolysates and permeates were assessed using a cyclooxygenase inhibition assay, targeting human COX-2 and ovine COX-1 enzymes, as described previously. COX (Prostaglandin H synthase) inhibition plays a positive role in the prevention of a variety of health conditions, and COX inhibitors have documented analgesic, anti-inflammatory, and antipyretic properties. They can influence pain and inflammation related to diseases like osteoarthritis and rheumatoid arthritis, musculoskeletal injury, migraines, and cancer. COX inhibitors are indicated for mild pain and are usually prescribed for short durations of time to patients. Generated hydrolysates were screened at concentrations of 1 mg/mL for their ability to inhibit COX-1 and COX-2 using the human COX-2 enzyme and ovine COX-1 enzyme and by measuring the peroxidase activity colour metrically using a spectrophotometer, and the appearance of N,N,N’,N’-tetramethyl-p-phenylenediamine (TMPD) at 590 nm in the presence and absence of the hydrolysate and a positive control (Resveratrol—COX-1) and X (COX-2) assayed at recommended concentrations in a 96 well plate format using the assay kit—COX colourimetric inhibition screening assay kit (Item No. 701050) from Cayman Chemicals (Cayman chemicals, Ellsworth Rd., Ann Arbor, MI, USA). Resveratrol (Sigma, Dublin, Ireland) is the positive control for COX-1 inhibition. COX inhibition was calculated using the following Equation:

% COX inhibition = (Corrected 100% initial activity − Corrected Inhibitor activity)/(Corrected 100% initial activity) × 100(1)

where Corrected 100% initial activity was the absorbance value recorded for wells containing 150 µL 1× assay Buffer, 10 µL of either COX-1 or COX-2 enzyme and 10 µL of solvent (DMSO) minus the absorbance value recorded for the background well containing 160 µL of 1× Assay Buffer and 10 µL of solvent.

#### 3.5.2. Monoacylglycerol Lipase (MAGL; EC 3.1. 1. 23) Inhibition

Monoacylglycerol lipase (MAGL) is a serine hydrolase that catalyses the hydrolysis of monoglycerides into glycerol and fatty acids and links the endocannabinoid and eicosanoid systems through degradation of 2-arachidaoylglycerol to arachidonic acid, a precursor of prostaglandins and other inflammatory mediators. MAGL inhibitors can have anti-nociceptive, anxiolytic, anti-inflammatory and even anti-cancer benefits. The Cayman’s Monoacylglycerol lipase inhibition screening assay kit was used to measure the inhibition of MAGL by generated hydrolysates and the positive control JZL 195 inhibitor. The assay was performed in compliance with the manufacturer’s instructions. MAGL hydrolyses 4-Nitrophenylacetate, resulting in a yellow product, 4-nitrophenol, that has an absorbance between 405–412 nm. The disappearance of the yellow colour is indicative of MAGL inhibition. MAGL inhibition was calculated using the following Equation:

% MAGL inhibition = (corrected 100% initial activity − corrected inhibitor activity)/(corrected 100% initial activity) × 100(2)

where corrected 100% initial activity was the absorbance value recorded for wells containing 150 µL 1× assay Buffer, 10 µL of MAGL enzyme and 10 µL of solvent minus the absorbance value recorded for the background well containing 160 µL of 1× Assay Buffer and 10 µL of solvent (1.4% DMSO in water).

#### 3.5.3. ACE-I Inhibition Assay

This assay was performed independently on Irish hydrolysates generated at Teagasc and Spanish hydrolysates generated by AZTI and assayed by the Leitat Technological Centre, using an ACE-I inhibitor assay kit and in accordance with the manufacturer’s instructions (ACE Kit—WST assay kit from Dojindo Laboratories, Rockville, MD, USA). All fractions were assayed at a concentration of 1 mg/mL HPLC grade water in triplicate and means and SD were calculated. The known ACE-I inhibitor Captopril was used as a positive control at a concentration of 1 mg/mL. Absorbance was measured with a FLUOstar Omega microplate reader (BMG LABTECH GmbH, Ortenberg, Germany) at 450 nm.

#### 3.5.4. DPP-IV Inhibition Assay

This assay was carried out using a DPP-IV inhibitor screening assay kit and in accordance with the manufacturer’s instructions (Cayman Chemicals, Ann Arbor, MI, USA). All hydrolysates (Irish, Spanish and Norwegian) were assayed in triplicate and means and SD were calculated. The known DPP-IV inhibitor Sitagliptin (Merck, Dublin, Ireland) was used as a positive control. Fluorescence intensity was recorded with a FLUOstar Omega microplate reader (BMG LABTECH GmbH) using an excitation wavelength of 355 nm and an emission wavelength of 460 nm.

#### 3.5.5. Antioxidant Capacity: ABTS Assay

The ABTS (2,2-azinobis-(3-ethylbenzthiazoline-6-sulfonic acid) assay adjusted to microplate volume was used to determine the antioxidant capacity of Spanish mesopelagic hydrolysates. The colourimetric results were measured in Varioskan™ LUX multimode microplate reader (Thermo Fisher Scientific, Waltham, MA, USA). In brief, 7 mM ABTS solution and 2.45 mM potassium persulfate were diluted in PBS (phosphate-buffered saline) for an absorbance of approximately 0.7 at 734 nm. The determination consisted in the decrease in absorbance at 734 nm of the reagent solution, 6 min after the sample was added in the micro-well in a ratio of 1:100 (*v*/*v*) (sample: ABTS solution), as the result of the reduction of the radical coloured ABTS. Trolox was used as the standard, and the antioxidant capacity (AOC) was calculated as Trolox Equivalent Antioxidant Capacity (TEAC, µM TE/mg of sample).

#### 3.5.6. Renin Inhibition Activity

Renin Inhibitory activity of hydrolysates was carried out according to the manufacturer’s instructions and as described previously [29]. The known renin inhibitor, Z-Arg-Arg-Pro-Phe-His-Sta-Ile-His-Lys-(Boc)-OMe, was the positive control. Percentage inhibition was calculated using the following Equation:

Renin inhibition % = 100% initial activity AF − inhibitor activity AF/100% initial activity AF × 100(3)

where AF is the average fluorescence. Initial activity is the assay performed without the presence of an inhibitor.

### 3.6. Peptides Identified Using Mass Spectrometry

The most active hydrolysates identified using in vitro bioactivity assays included the Spanish *Maurolicus muelleri* hydrolysate generated with Alcalase 2.4 L FG (MME02) and the Spanish *Maurolicus muelleri* hydrolysate generated with endogenous enzymes (MMC019). Additionally, the Irish *Maurolicus muelleri* hydrolysed with Alcalase (23) displayed MAGL inhibitory activity. These hydrolysates were selected for subsequent mass spectrometry (MS) analysis. The 3-kDa MWCO permeate fractions generated from each bioactive hydrolysate were processed for mass spectrometry analysis using the Preomics Phoenix Clean-up Kit (96×) (Preomics, D-82152 Planegg/Martinsried, Planegg, Germany) in accordance with the manufacturer’s instructions and as described previously for microalgal peptides [30]. Peptides were identified with a confidence of ≥ 95%.

### 3.7. Assessment of Bioactive Potential of Identified Peptides

The bioactive potential of each peptide was predicted using several in silico/AI methods, some of which were described previously by this group [48]. Briefly, the Peptide Ranker tool (http://bioware.ucd.ie/~compass/biowareweb/ [49], accessed on 22 January 2024) and PreAIP (http://kurata14.bio.kyutech.ac.jp/PreAIP/about.php) (accessed on 23 January 2024) [50], were used to assess overall bioactivity and potential of the peptides to act as anti-inflammatory molecules. The novelty of identified peptides was determined by performing a literature and database search in BIOPEP-UWM (http://www.uwm.edu.pl/biochemia/index.php/en/biopep [51], accessed on 23 January 2024). The umami activity of selected peptides identified using mass spectrometry was assessed using Umami-MRNN https://umami-mrnn.herokuapp.com/ (accessed on 23 January 2024) [29] and BIOPEP-UWM as described previously [50].

## 4. Conclusions

Results generated in this work corroborate the potential of mesopelagic fish as a source of nutrients but additionally as a reservoir for novel, bioactive peptide generation. Several unique, novel bioactive peptides were identified, and their anti-inflammatory and heart health potential was validated using in silico and in vitro bioassays. Additionally, the Umami flavour potential of generated hydrolysates was assessed using in silico analysis of specific peptides. The bioactivities of generated hydrolysates are promising and offer the potential for use in the treatment of inflammatory health conditions like psoriasis and others and may be commercially exploitable; however, further in vivo work is required. The work demonstrates the potential of mesopelagic fisheries as a resource for the generation of new, novel bioactive compounds for food and pharma applications.

## Figures and Tables

**Table 1:** Peptide sequences identified from mesopelagic fish species hydrolysates generated using different enzymes, including Alcalase, and in silico analysis predicted bioactivity results for selected peptides identified using mass spectrometry using different programmes, including PeptideRanker http://bioware.ucd.ie/~compass/biowareweb/, accessed on 30 September 2023; PreAIP http://kurata14.bio.kyutech.ac.jp/PreAIP/, accessed on 11 December 2023; BIO-PEP-UWM https://biochemia.uwm.edu.pl/biopep-uwm/, accessed on 6 January 2024; AntiDMPpred available at http://i.uestc.edu.cn/AntiDMPpred/cgi-bin/AntiDMPpred.pl, accessed in January 2024 and Umami-MRNN https://umami-mrnn.herokuapp.com/, accessed in January 2024.

Hydrolysate	Peptide Sequence	Peptide Ranker Value	PreAIP RF Combined Values	Anti-Diabetic Prediction (AntiDMPpred)	BIOPEP-UWM	Umami
CE21009 Haul 23 *Maurolicus muelleri* (Code 23) Alcalase hydrolysate. Irish sample. Freeze-dried. High Confidence AIP (0.482).	KTLRKMGKWCCHCFPCCRGSGKSNVGAW	0.999	High confidence AIP (0.731)	Low probability	Novel	umami, predicted threshold: 25.47 mmol/L
	DGINVLGLIVFCLVLGIVIGRKWEKGQIL	0.996	High confidence AIP (0.560)	Low probability	Novel	umami, predicted threshold: 12.65 mmol/L
Origin—Thin-lipped Mullet	FDAFLPM	0.955	Medium confidence AIP (0.392)	Low probability	Novel	Non-umami
	GLGGMLF	0.939	Low confidence AIP (0.370)	Low probability	Novel	umami, predicted threshold: 35.46 mmol/L
Origin—*Salmo salar*—Atlantic Salmon	QCPLHRPWAL	0.932	High confidence AIP (0.499)	Low probability	Novel	Non-umami
	LACNCNLHARRCRFNM	0.908	High confidence AIP (0.629)	Low probability	Novel	umami, predicted threshold: 37.66 mmol/L
	TFSWGFDDFSCC	0.889	High confidence AIP (0.496)	Low probability	Novel	umami, predicted threshold: 14.82 mmol/L
	GINVLGLIVFCLVLGI	0.888	High confidence AIP (0.620)	Low probability	Novel	umami, predicted threshold: 35.08 mmol/L
	LLSSELQSLLIATTCLRELISCC	0.873	High confidence (0.614)	Low probability	Novel	umami, predicted threshold: 13.25 mmol/L
Origin—*Makaira nigricans*—Atlantic Blue Marlin	NVGEVVCIFLTAALGLPEALI	0.868	High confidence AIP (0.612)	Likely to be anti-diabetic (probability of 0.8)	Novel	umami, predicted threshold: 19.89 mmol/L
*Maurolicus muelleri* (MMC019) Endogenous enzyme autolysis (Spanish sample), spray dried. High confidence AIP (0.514)	SFVPNGASLEDCHCNLPCLA	0.874	High confidence AIP (0.506)	Low probability	Novel	umami, predicted threshold: 30.65 mmol/L
	GFSAVNMRKFG	0.797	High confidence AIP (0.527)	Low probability	Novel	umami, predicted threshold: 30.85 mmol/L
Origin—*Cypinus carpio*—Common Carp	IAGFEIFDFNSLEQLC	0.734	High confidence AIP (0.540)	Low probability	Novel	umami, predicted threshold: 36.00 mmol/L
	NLFKDCNF	0.693	Medium confidence AIP (0.467)	Low probability	Novel	umami, predicted threshold: 18.79 mmol/L
	PFGAADQDPF	0.677	Low confidence AIP (0.370)	Low probability	Novel	Non-umami
	NSGAGILPSPSTPRFP	0.621	Medium confidence AIP (0.453)	Low probability	Novel	umami, predicted threshold: 25.95 mmol/L
	DVEFLPPQLPSDKFKDDPVG	0.601	Medium confidence AIP (0.433)	Low probability	Novel	umami, predicted threshold: 20.45 mmol/L
Origin—*Takifuga rubipes*—Japanese Puffer Fish	GFAGDDAPR	0.598	Negative AIP (0.284)	Low probability	Novel	umami, predicted threshold: 1.68 mmol/L
	FSPFGAAD	0.58	Low confidence AIP (0.346)	Low probability	Novel	umami, predicted threshold: 17.39 mmol/L
	PSRILYG	0.574	Medium confidence AIP (0.412)	Low probability	Novel	Non-umami
*Maurolicus muelleri* (MME02—Spanish haul) (medium confidence AIP—0.446)	VFIPFNPL	0.871	Low confidence AIP (0.382)	Low probability	Novel	Non-umami
	NDLPWEF	0.861	Low confidence AIP (0.349)	Low probability	Novel	Non-umami
	VLLFFYAPWCGQ	0.846	High confidence AIP (0.524)	Low probability	Novel	Non-umami
	CGRASCPVLCSG	0.845	High confidence AIP (0.480)	Low probability	Novel	umami, predicted threshold: 23.60 mmol/L
Origin—*Makaira nigricans*—Atlantic Blue Marlin	GFNPPDLDIM	0.828	Low confidence AIP (0.382)	Low probability	Novel	non-umami
Origin—*Cypinus carpio*—Common Carp	SDNAYQFMLT	0.72	Medium confidence AIP (0.412)	Low probability	Novel	umami, predicted threshold: 34.06 mmol/L
	CLGSPNPLDII	0.687	Medium confidence AIP (0.408)	Low probability	Novel	umami, predicted threshold: 36.98 mmol/L
	RCPEALF	0.672	High confidence AIP (0.556)	Low probability	Novel	non-umami
	ADDEDADGESSGEPPGAPKQEEAI	0.667	High confidence AIP (0.469)	Low probability	Novel	umami, predicted threshold: 8.32 mmol/L
	DSFGRLT	0.662	Low confidence AIP (0.387)	Low probability	Novel	umami, predicted threshold: 12.87 mmol/L

## Data Availability

Further information is available on results from the MEESO database or directly from the corresponding author. All data is open access and available from the corresponding author.
